# Foraging Decisions in Risk-Uniform Landscapes

**DOI:** 10.1371/journal.pone.0003438

**Published:** 2008-10-17

**Authors:** Jana Anja Eccard, Thilo Liesenjohann

**Affiliations:** 1 Animal Behavior, University of Bielefeld, Bielefeld, Germany; 2 Animal Ecology, University of Potsdam, Potsdam, Germany; University of Exeter, United Kingdom

## Abstract

Behaviour is shaped by evolution as to maximise fitness by balancing gains and risks. Models on decision making in biology, psychology or economy have investigated choices among options which differ in gain and/or risk. Meanwhile, there are decision contexts with uniform risk distributions where options are not differing in risk while the overall risk level may be high. Adequate predictions for the emerging investment patterns in risk uniformity are missing. Here we use foraging behaviour as a model for decision making. While foraging, animals often titrate food and safety from predation and prefer safer foraging options over riskier ones. Risk uniformity can occur when habitat structures are uniform, when predators are omnipresent or when predators are ideal-free distributed in relation to prey availability. However, models and empirical investigations on optimal foraging have mainly investigated choices among options with different predation risks. Based on the existing models on local decision making in risk-heterogeneity we test predictions extrapolated to a landscape level with uniform risk distribution. We compare among landscapes with different risk levels. If the uniform risk is low, local decisions on the marginal value of an option should lead to an equal distribution of foraging effort. If the uniform risk is high, foraging should be concentrated on few options, due to a landscape-wide reduction of the value of missed opportunity costs of activities other than foraging. We provide experimental support for these predictions using foraging small mammals in artificial, risk uniform landscapes. In high risk uniform landscapes animals invested their foraging time in fewer options and accepted lower total returns, compared to their behaviour in low risk-uniform landscapes. The observed trade off between gain and risk, demonstrated here for food reduction and safety increase, may possibly apply also to other contexts of economic decision making.

## Introduction

Ecological theory assumes, that animals have adapted to their environment by optimising behavior in order to maximise fitness. Foraging behavior is often used as a paradigm of optimal behavior [1]. While foraging, a forager may itself become prey to another forager and it is pivotal to reduce the risk of being killed or seriously injured by predation to increase the forager's fitness. Foragers should thus not only maximise their gain but also minimize predation risk by making decisions on where to forage and when to leave a patch [2], [3].

Antipredatory adaptations to foraging behavior have largely been studied in risk-heterogeneous environments, such as desert ecosystems with a choice of microhabitats [4] or in habitats deliberately made risk-heterogeneous, for example by mowing [5]. In such situations foragers value patches in safer locations higher than in unsafe locations, which can be measured by the quitting harvest rate [6]. Meanwhile, many environments are relatively uniform in their risk distribution, i.e. predation risk is evenly spread over space from the prey's perspective and all patches are equally unsafe. Environments can be risk uniform by their uniform structure, or risk uniform independent of habitat structures if the predator matches body size and locomotive ability of the prey and prey can thus not hide from predation. Further, risk uniformity may occur if predators follow an ideal free distribution in relation to prey availability in differently structured habitats so that the per capita predation risk for the prey individuals is equal across differently frequented habitats. Indeed IFD theory would suggest that risk uniformity should be very common in natural systems [7].

In this paper we will introduce predictions for risk-uniform landscapes and discuss the role of opportunity costs and travelling risk. We will then present data from experimental foragers in artificial, risk-uniform landscapes under controlled laboratory conditions that support our predictions.

### Risk-uniform landscapes

When comparing between risk-uniform landscapes we have to compare between environments. Such between-environment comparisons were done earlier to assess the importance of mean resource level [8] including mean fitness value of an environment [9], however, predation risk in these models/experiments varied among patches within the environment. We here compare among environments with different risk levels that have a uniform resource distribution and a uniform risk distribution among the patches within the environment.

**Figure 1:**
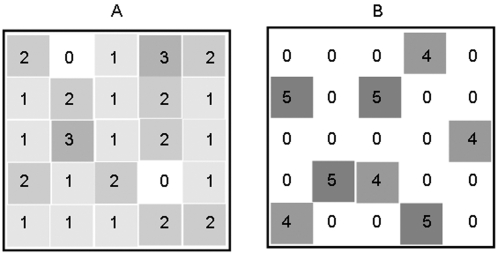
Predicted distribution of total investment. The same total investment (sum of cell values) into a resource landscape with (A) uniformly low risk where investment is evenly spread among options or (B) uniformly high risk with distinct investment peaks (B). Higher number and darker colour indicate a higher local investment.

We base our considerations on the marginal value theorem (MVT, [10]) with depletable resources that are depressed by their exploitation. Foragers should leave a patch as soon as the return rate from this resource drops below the average return rate of the habitat. Here we extrapolate this local decision rule to a conceptual landscape consisting of many depletable options. The investment pattern resulting from many local decisions by a forager switching among options would be evenly distributed among the options (Fig. 1A). Since the mean return rate of the habitat decreases with depletion, the even distribution of investment patterns over time should in theory slowly drain the resource level of the habitat.

With an extension of the MVT it is possible to include the local predation risk into optimality models on patch depletion [2], stating that a forager should leave each resource patch when the return rate is no longer greater than the sum of the energetic costs, predation costs, and missed opportunity costs (MOCs) of foraging. Predictions follow, that if two patches share the same energetic cost of foraging and the same return rate function, then any differences in patch residency should reflect differences in patch specific risks of predation. Consequently, measurements of patch residency are used to compare predation risks among patches [2], [11], and can be used to map the forager's perception of differences in predation risk across landscapes [12].

Applying Brown's model [2] to an environment where metabolic costs of foraging and the predation risk is constant among patches, a forager could also base its local decision on patch residency on the MOCs inherent to the forager in his environment. The MOCs include fitness costs of not engaging into other, fitness- increasing alternative activities such as mate search, territory defence or recovery and are constant for any forager in a given environment. Among environments though, MOCs depend on habitat richness, but also on feeding in and travelling between patches and on the survival rate in the habitat [8], [13]. Thus, MOCs also have a predation risk component. We therefore predict that if predation risk is uniformly low, animals will engage in other activities than foraging. The patch residency, with constant costs of foraging and constant predation risk among options should then reflect the costs of missed opportunities. Both quitting return rate and missed opportunity costs in low risk contexts therefore are high, which is reflected in an early and frequent leaving of patches. This prediction differs from predictions and observations in risk-heterogeneous environments, where locally relatively low predation risk results in low local quitting harvest rates [2], [11]. If we extrapolate our prediction of frequent leaving and use of many patches to a risk-uniform landscape level, variance in foraging effort among patches will be low and animals' local decisions will create foraging landscapes with an even distribution of foraging effort.

If risk is uniformly high, exploration of new food sources as well as social and sexual activities make potential prey animals conspicuous and vulnerable and thus increase predation risk. Animals should therefore invest little in alternative activities and only conduct the absolute necessary minimum activity of foraging. Further, social or sexual interactions are reduced because conspecifics reduce activity too, due to the same high predation risk. If predators distribute themselves towards the availability of prey, as predicted from ideal free distribution [7] the predation risk affects all conspecifics equally. Opportunity costs of missing other activities are therefore low and reflected in low quitting return rates, i.e. a long local investment. This contradicts the effects of *local* high predation risk in risk-heterogeneity where quitting harvest rates are high with high local predation risk [2], [11]. Thus, high investment in single options under high uniform risk may increase disproportionately, increasing the variance in foraging effort among patches. Extrapolated to a risk-uniform landscape level, foraging effort results in a landscape with high and isolated peaks while other options remain untouched (Fig. 1B).

### Small mammals and risk-uniformity

The meaning of risk-uniformity depends on the size of the organisms, its mobility and the scale of microhabitats and habitat structures. For small mammals that we used as experimental foragers in our experimental system, agrobiomes with monocropping often exceed the scale of small mammal territories and can thus be risk-uniform in their avian predation risk. Small predators of rodents, such as snakes or mustelids match their rodent prey in size and locomotion type [14] and may distribute themselves according to prey availability in space and time [7]. Rodents cannot hide from such omnipresent predators, which therefore produce a uniformly distributed predation risk over the entire landscape. Some foraging patters produced in rodent-mustelid systems [15], [16] are difficult to understand within a risk-heterogeneous approach. In the study of Eccard et al. [16] in a large enclosure experiments, rodents increased their foraging in artificial food patches and depleted these to lower levels in the presence of mustelid predators than in the absence. Patterns can possibly be explained using the risk-uniform landscape approach to foraging we propose here.

## Methods

### Experimental foragers in risk-uniform landscapes

Foraging behaviour of 12 male bank voles (*Myodes glareolus*) was investigated in an artificial resource landscapes with uniformly spread predation risk. In a 9 m^2^ indoor arena covered with dry sand we offered 25 food patches (15 cm diameter, 0.2 g millet/0.4 l sand) in a 5×5 grid with 40 cm distance, and a central nest box. Ground cover was provided by a framed metal lattice (4 m^2^ with 1 cm×1 cm cells). We approached foraging decisions as the shape of a 3-dimensional investment landscape resulting from the (2-dimensional) position of a resource patch and the local investment into each patch defining the third dimension.

Each animal was tested in two temporally separated treatments under either uniformly safe or uniformly risky conditions. In the “safe” treatment we covered the foraging grid, potentially protecting the forager from attacks by birds of prey. In the “risky” treatment no ground cover was provided. Attacks by birds of prey are unforeseeable and unavoidable, for most prey species the avoidance of open ground is an apparently invariant result in foraging studies with antipredatory behaviour of rodents [11]. Thus, even without a hawk in the research hall the subjective perception of predation risk is higher without cover than with cover for a ground dwelling mammal. Each individual was subjected to both risk levels for 2 days each, allowing pair-wise comparisons within individuals among risk levels. Animals were divided into two groups with reversed order of treatments in the two experimental phases. Phases lasted for 2 subsequent days in which data were gathered during 6 evening hours, with 4 days habituation to the arena and a 2–6 day break between phases.

**Figure 2:**
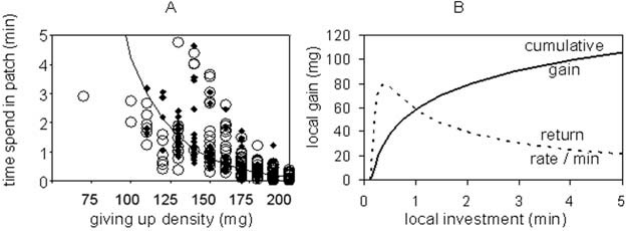
Local gain and investment (patch residency). (A) Observational data on patch residency from a video analysis of foraging bank voles (11 animals, 22 trials, 369 experimental patches with initial food density of 200 mg, open circles: safe risk-uniform conditions, diamonds: risky uniform conditions) and predictions (line) of patch residency by the measured amount of food left over in the patch (giving up density) with an exponential model: time = −272 sec*e(−7327,6*gud(mg)) with R2 = 0.571, F = 491; p<0,001. (B) Predictions for cumulative gain (solid line) and diminishing returns (rate per minute, dashed line) over patch residency based on A.

From both experimental phases we obtained depletion levels of food for all patches. During the second experimental phase animals were filmed to establish the relation of patch residency (invested time) and depletion levels (Fig. 2A). Local patch use followed the classical patterns [10] of diminishing returns with increasing patch residency (Fig. 2B).

**Figure 3:**
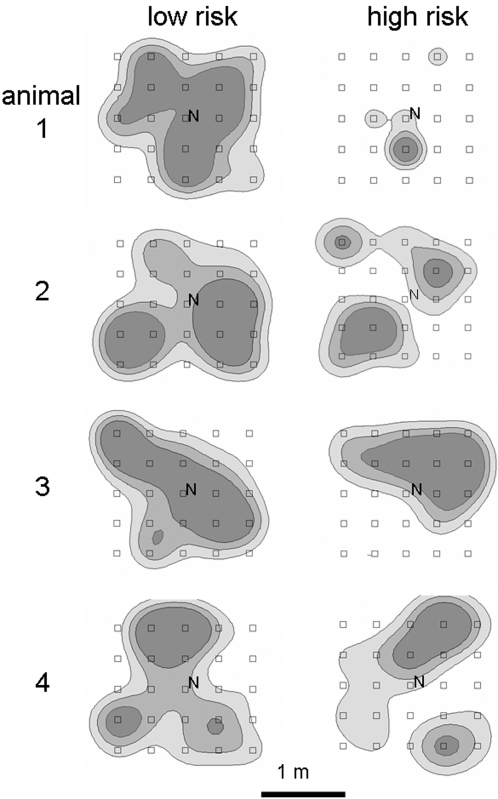
Spatial distribution of time investment. Four exemplary foragers (male bank voles) at their first day of foraging under safe (left) and risky (right) conditions. Investment is displayed as Kernel contour cores 50% (darkest), 75% (lighter) and 95% (lightest shade) based on location density (time investment) and distance between locations.

We analyzed spatial distribution of the invested time over the entire resource landscape with RangesVI, a GIS-based location analysis program [17] and determined the number of patches where 50% of the time was spent (cluster analysis). Choice of percentage for cores was arbitrary. Similar results as presented for 50% cores were also obtained for 30% and 80% cores. Spatial clumping of investment was calculated relative to distance between locations (Kernels contour analysis, Fig. 3).

Preliminary analysis with repeated measures ANOVA models revealed, that day (2 days per treatment) as inner subject repeats, treatment order (2 levels, between-subject factor), progressing season (covariate) and weight loss during experiment (covariate) were not explaining variance. We therefore concluded the analysis using within-animal means of the two days for each of two risk treatments, and compared among risks with paired t-tests within subjects where appropriate (tests for normality and homogeneity of variances) and non-parametric Wilcoxon tests otherwise.

One may argue, that artificial landscapes were not truly risk uniform in their distance to safety, i.e. either central nest box or the arena wall. We therefore analysed the proportional use of the 9 patches near the central nest (their preference would be indicated by proportional values >1) in relation to the 16 patches closer to the arena walls (their preference would be indicated by proportional values <1).

Ethical note: Animals were captured by live trapping from premises of the University of Bielefeld (Germany) and were returned after the experiment to the capture grid. Animal care and housing complied with institutional guidelines. Permissions by Umweltamt Bielefeld (360.12.06.01.3) and Gesundheits-, Veterinär- und Lebensmittelüberwachungsamt Bielefeld (530.42).

## Results

Experimental foragers (n = 12) invested similar length of time in foraging into the resource landscapes in both risk treatments (690±376 (mean±SD) seconds in uniform high risk and 660±399 seconds in uniform low risk; Wilcoxon signed rank test, Z = −0.2, p = 0.875). Meanwhile, the total amount of food removed from the experimental grid was lower under uniform high risk (0.51±0.25 g) than under uniform low risk conditions (0.72±0.29 g, t = 2.5, p = 0.028).

Individuals' gain function per patch did not differ among risk treatments (ANCOVA of GUDs by residence time (data of Fig. 2A) with no interaction of risk(factor) and time(covariate, log transformed): F interaction (1, 355) = 1.4, p = 0.237, individual was added as a random factor, F: risk treatment (1, 355) = 2.9, p = 0.088; F log(time) = 542.8, p<0.001).

In the high risk treatment, less patches were used for foraging: 50% of foraging time was invested into 24%±12% of patches under high risk and into 40%±20% under low risk conditions (paired t-test, t = 5.6, p = 0.0001). This resulted in a smaller area of investment cores under high risk (0.35±0.15 m^2^) than under low risk (0.62±0.15 m^2^, t = 2.6, p = 0.023, with similar number of cores: risky: 1.6±0.6 cores, safe 1.6±0.5 cores, t = 0.0, p = 1.0, Fig. 3).

There were no behavioural indicators that locations differed in perceived local risk relative to safety. Proportional use of patches closer to the nest or to the wall did not differ from 1, one-sample t-test with n = all 24 trials, t = 1.1, p = 0.281; and with n = 12 trials in high risk treatment, t = 0.97, p = 0.354).

## Discussion

In summary, under uniformly high risk animals invested the same foraging time into fewer patches as under low risk conditions and, by exploiting these with diminishing returns over time, accepted lower total returns from the landscape. The experimental results support our predictions for distinct investment patterns between levels of uniform risks. Predictions may further be supported by our results from field experiments with rodents under a uniform mustelid predation risk [16] where artificial food patches were exploited to lower levels in the presence of weasels than in their absence.

In our experiment reported here, foragers under uniform, low risk behaved as predicted by the marginal value theorem on diminishing returns [10] and distributed their effort as to maximise their returns. Foragers under uniform high risk concentrated their effort to very few options and exploited these consequently (Figure 3) despite diminishing returns. This behaviour minimizes the risk associated with other activities outside feeding in the chosen location which are probably also characterized by increased predation risk. With other activities reduced, their missed opportunity costs are devaluated. With its strategy of distribution of investment, the animal potentially depresses the overall return from the resource landscape. This general trade off between gain and risk, demonstrated here for reduced food gain and increased safety from predation, may possibly apply also to other economic decision contexts in biology, psychology or economy with uniform risk distributions.

Alternative explanation for our results in the riskier treatment could be an increased risk in travelling among patches (1) or an increased vigilance while foraging (2). First, the risk of predation may function to increase the danger while switching patches, compared to a continuation of foraging in the current patch. In a natural habitat with large distances between patches this explanation may hold – animals may leave scent trails or produce noise while traveling, or by moving increase the chance to encounter a predator. In the small scale of the experimental landscape the explanation can be refuted, since distances between patches were short relative to the size of patches and relative to the forager's mobility. Our data further showed that foragers did not prefer the patches closer to safety, another indication for that distance between patches was not relevant at the investigated scale. Furthermore, risk treatment (cover or open) was the same for the patches and the matrix between them in our experiment so that risk should not differ among feeding and travelling. As a second alternative explanation, vigilance during foraging in the riskier treatment could have reduced the gain function. In our analysis of gain per patch (Fig. 2a) we could not find a difference in the gain function between the risk treatments and therefore have little support for this alternative explanation.

In conclusion, high risk levels in a risk-uniform landscape can produce foraging patterns different from low risk levels. Further, patterns cannot be directly anticipated from theory based on risk-heterogeneity. Thus, decisions in uniform risk should be included in a comprehensive theoretical framework on foraging decisions under predation risk.
